# High-altitude retinopathy - a potential model for retinal haemorrhages in suspected shaken baby syndrome

**DOI:** 10.1038/s41433-025-03824-z

**Published:** 2025-05-02

**Authors:** Niels Lynøe, Anders Eriksson

**Affiliations:** 1 Centre for Healthcare Ethics, Karolinska Institutet, Stockholm, Sweden; 2 Department of Clinical Sciences, Forensic Medicine, Umeå University, Umeå, Sweden

**Keywords:** Anatomy, Medical research

## Background

In his original publication from 1972 [1], the American radiologist John Caffey suggested that bilateral retinal haemorrhages (RH) are potentially pathognomonic of violent shaking – later referred to as shaken baby syndrome (SBS). He also claimed that there is a striking lack of injuries such as signs of head trauma [1]. Caffey assumed that these infants and children were shaken as he – in the absence of injury - found no other plausible explanation for the infant’s collapse [2]. In 2009, SBS was conflated into the heterogeneous group of Abusive Head Trauma (AHT) [3], which since has created confusion regarding terminology when talking about the SBS subgroup [4].

A systematic review *“confirmed”* that *“intraocular haemorrhages in infants - particularly bilateral, extensive, and multilayered - are highly specific for AHT”* [5], and another review “*confirmed*” that certain patterns of RH were far commoner in AHT than in accidental trauma, but that no retinal findings were unique to AHT [6]. However, both these systematic reviews [5, 6] rely on single studies which suffer from circular reasoning, resulting in high risk of bias and hence having a very low scientific quality [7].

More recent investigations suggest, however, that the clinical findings (“the triad”) widely considered to be indicative of shaking (RH, subdural haemorrhage (SDH), and brain oedema or symptoms of encephalopathy) are instead markers of the degree of intracranial pathology [8, 9], and that previous results were driven by selection effects [8]. The conclusion was that RH are secondary and non-specific, which is supported also by a previous study [10]. The possible specificity of RH for SBS/AHT is however one thing, the pathogeneses of RH and subdural haemorrhage (SDH) is something else. Without understanding the pathogenetic processes behind these medical findings, *“it remains impossible, despite the assertions of some authors, to be certain that all infants demonstrating them have been the victims of attempted, or actual, murder”* [11].

Accordingly, if the assumption that certain types or numbers of RH are common in suspected SBS cases - or even *specific* to SBS - is based on insufficient evidence [7], it is relevant to examine *any* plausible mechanism, also new theories including the so-called hypoxia theory. Particularly so, when this theory was found to be more plausible than the traditional SBS theory [12], and if medical findings alone are allowed to constitute evidence of a serious crime.

### Hypobaric hypoxia effects in adults – an analogy?

*In infants*, neuropathological studies of brains from cases of suspected SBS did not confirm the traditional SBS theory predicting diffuse axonal injury [13]. Instead, brain hypoxia only was demonstrated in cases without signs of trauma, and hypoxia was suggested as the cause of triad findings [13, 14]. The mechanism was hypothetically described as a cascade reaction resulting from a prolonged spontaneous breathing stop due to the immature brain causing brain hypoxia and oedema, increased intracranial pressure, and blood oozing from small vessels, followed by RH and SDH [14–17].

*In adults*, well-known effects of hypobaric hypoxia include acute mountain sickness, high-altitude retinopathy, and brain oedema - potentially lethal, just like isolated triad findings in infants. Acute mountain sickness occurs - if not acclimatized - during the first days at an altitude of ≥2500 m. Lower oxygen saturation, higher ascent, and longer duration at high altitudes correlated with more RH [18]. Hypoxic damage to the vascular endothelium of small retinal vessels with oozing of blood was suggested to cause RH [19, 20], after a definite time lag [19].

It has been claimed that acute hypoxia alone is insufficient to cause SDH and RH, and that “(*a)cute* hypoxia resulting from transient apnoea has not been shown to result in the SBS picture” [21]. The question is, however, not what effects *acute* hypoxia may or may not have, but what effects *prolonged* episodes of asphyxia can have [16, 22].

This possible mechanism has not been investigated despite a case of SDH associated with acute mountain sickness was presented already in 2012. After a stay at an altitude of >2700 m, this young man developed during the following days progressive headache and sudden deterioration of consciousness. Bilateral SDH were detected and treated with craniotomies and drainage. No head trauma, no coagulopathy, nor any other accepted cause of SDH was present, and recovery was rapid [18]. Obviously, hypobaric hypoxia may have a progressive, prolonged development even days after exposition, and all triad components can develop in an adult after exposition to hypoxia.

Of relevance for a possible analogy is that studies of infants indicate that RH are associated with intracranial pathology and raised intracranial pressure [8, 9]. Further, the development of medical findings seems to be delayed both in infants and in hypobaric retinopathy [18, 21], compatible with a gradual development of the suggested hypoxia cascade. In fact, there are several possible common denominators between RH (and SDH) in suspected SBS and mountain sickness (Table 1).

**Table 1:** Possible common denominators and understanding between the mechanism of RH (and SDH) in suspected SBS cases (among infants) and acute mountain sickness (among adults).

Possible common denominators	Suspected SBS	Mountain sickness
Cerebral oedema	Present	Present
Increased intracranial pressure	Present	Present
Gradual development of symptoms	Yes	Yes
RH (and SDH) can be associated with raised intracranial pressure	Yes	Yes
Prolonged episodes of hypoxia might cause RH (and SDH)	Yes, probably	Yes, probably
Retinal vessel walls damaged by hypoxia	Yes	Yes
Risk of fatal outcome	Yes	Yes

## Concluding suggestions

There are several controversies about the mechanism causing the development of RH in suspected SBS, and also about a valid reference standard for diagnosing SBS. We suggest that comparisons of RH in suspected SBS and RH in acute mountain sickness could help understanding the pathophysiology behind RH in alleged SBS cases. This could represent an interesting strategy to illuminate the possible role of hypoxia as a cause of RH, as well as the gradual development of RH and SDH and the possible significance of disc oedema. Even though caution must be applied when comparing conditions among infants versus among adults, we suggest that concerned researchers seek common understanding on which to proceed and maybe identify an auxiliary hypothesis regarding under which conditions hypoxia might cause RH (and SDH) in young infants.

In summary, we suggest that the hypoxia mechanism theory in cases of suspected SBS should be compared with high altitude hypobaric retinopathy to gain better understanding of the pathogenesis of RH in suspected SBS.
